# Evaluation of a multidisciplinary lipid clinic to improve the care of individuals with severe lipid conditions: a RE-AIM framework analysis

**DOI:** 10.1186/s43058-021-00135-8

**Published:** 2021-03-19

**Authors:** Laney K. Jones, Megan McMinn, David Kann, Michael Lesko, Amy C. Sturm, Nicole Walters, Nan Chen, Kerrianne Fry, Ross C. Brownson, Samuel S. Gidding, Marc S. Williams, Alanna Kulchak Rahm

**Affiliations:** 1 Genomic Medicine Institute, Geisinger, Danville, PA USA; 2 Center for Pharmacy Innovation and Outcomes, Geisinger, Danville, PA USA; 3 Heart Institute, Geisinger, Danville, PA USA; 4 Department of Population Health Sciences, Geisinger, Danville, PA USA; 5 Prevention Research Center in St. Louis, Brown School at Washington University in St. Louis, St. Louis, USA; 6 Department of Surgery, Division of Public Health Sciences, Alvin J. Siteman Cancer Center, Washington University School of Medicine, Washington University in St. Louis, St. Louis, USA

**Keywords:** RE-AIM, Hyperlipidemia, Cardiology, Implementation science

## Abstract

**Background:**

Individuals with complex dyslipidemia, or those with medication intolerance, are often difficult to manage in primary care. They require the additional attention, expertise, and adherence counseling that occurs in multidisciplinary lipid clinics (MDLCs). We conducted a program evaluation of the first year of a newly implemented MDLC utilizing the RE-AIM (reach, effectiveness, adoption, implementation, and maintenance) framework to provide empirical data not only on program effectiveness, but also on components important to local sustainability and future generalizability.

**Methods:**

The purpose of the MDLC is to increase the uptake of guideline-based care for lipid conditions. Established in 2019, the MDLC provides care via a centralized clinic location within the healthcare system. Primary care providers and cardiologists were invited to refer individuals with lipid conditions. Using a pre/post-study design, we evaluated the implementation outcomes from the MDLC using the RE-AIM framework.

**Results:**

In 2019, 420 referrals were made to the MDLC (reach). Referrals were made by 19% (148) of the 796 active cardiology and primary care providers, with an average of 35 patient referrals per month in 2019 (SD 12) (adoption). The MDLC saw 83 patients in 2019 (reach). Additionally, 50% (41/82) had at least one follow-up MDLC visit, and 12% (10/82) had two or more follow-up visits in 2019 (implementation). In patients seen by the MDLC, we found an improved diagnosis of specific lipid conditions (FH (familial hypercholesterolemia), hypertriglyceridemia, and dyslipidemia), increased prescribing of evidence-based therapies, high rates of medication prior authorization approvals, and significant reductions in lipid levels by lipid condition subgroup (effectiveness). Over time, the operations team decided to transition from in-person follow-up to telehealth appointments to increase capacity and sustain the clinic (maintenance).

**Conclusions:**

Despite limited reach and adoption of the MDLC, we found a large intervention effect that included improved diagnosis, increased prescribing of guideline-recommended treatments, and clinically significant reduction of lipid levels. Attention to factors including solutions to decrease the large burden of unseen referrals, discussion of the appropriate number and duration of visits, and sustainability of the clinic model could aid in enhancing the success of the MDLC and improving outcomes for more patients throughout the system.

Contributions to the literature

- Multidisciplinary lipid clinics (MDLCs) lead to better guideline concordance, medical management, and patient outcomes for patients within a large healthcare system.
- Utilizing RE-AIM to analyze a clinical implementation helps to standardize reporting of outcomes.
- Limited capacity and resources for MDLCs may limit their ability to meet the patient demand within a large healthcare system.

## Background

The reduction of lipid levels has been shown to prevent cardiovascular disease (CVD) at individual and population levels [1]. Evidence-based guidelines for lipid therapy exist [2]. While primary care is sufficient for many, some individuals have medication side effects or underlying severe lipid conditions that make management more difficult. These individuals require specialized care in order to achieve lower cholesterol and triglyceride levels and the corresponding CVD risk reduction benefit.

Multidisciplinary lipid clinics (MDLCs) focus effort on reducing CVD risk for individuals with hyperlipidemia conditions that are complex and hence not easily controlled. Primary care providers (PCPs) refer complex patients to MDLCs with the expertise to more effectively manage medication options and diagnose underlying causes of severe dyslipidemia. While MDLCs may vary in staffing, typical clinical staff includes lipid specialist physicians, advanced practitioners, pharmacists, dieticians, and genetic counselors working in concert [3–9]. Successful deployment of evidence-based cholesterol guidelines in these complex patients requires several clinical skills: nutrition, to improve diet and explain differences in saturated versus unsaturated fats; diagnostic expertise regarding common and rare cholesterol disorders; and pharmacology, to focus on medication titration, management, and lifelong adherence. Such MDLCs have been shown to increase the number of individuals achieving target low-density lipoprotein cholesterol (LDL-C) goals and improve lipid-lowering medication adherence, effectively lowering the CVD risk of patients seen by the MDLC [3, 7].

MDLCs have not previously been evaluated using an implementation science framework [10]. Applying an implementation science framework can help standardize the reporting, examine barriers and facilitators necessary for a successful MDLC program, and evaluate implementation efforts within a healthcare system. Outcomes of the implementation science process would include identifying gaps in care missed by the referral process to the specialized clinic, improving sustainability, and developing generalizability to other healthcare systems. When there is an implementation gap, implementation science can help to understand the causes of the gap.

Reach, effectiveness, adoption, implementation, and maintenance (RE-AIM) is one framework that has been used for over 20 years in the planning and evaluation of interventions [11, 12]. For this study, we conducted an implementation science evaluation of the first year of a newly created MDLC utilizing the RE-AIM framework to provide empirical data not only on program effectiveness, but also on components important to local sustainability and future generalizability and replication.

## Methods

### Setting

Geisinger is an integrated healthcare system consisting of multiple hospitals, outpatient facilities, and a health plan located in 45 counties in central and northeast Pennsylvania. The system provides care for approximately 1.5 million patients annually. Clinical decisions and guidance for procedures and treatments are made by designated clinical teams, and full implementation is expected by every healthcare provider in the system. This method not only ensures consistency and high-quality care, but also promotes evidence-based care by reducing unexplained clinical variation. Additionally, coverage by the health plan is synchronized with clinical decisions made within the healthcare system to ensure high-quality care is affordable and accessible to all health plan members (about a third of Geisinger patients). Geisinger serves a rural, medically underserved, and low-income population. In Geisinger’s coverage area, 32 of the 45 counties are designated as rural, and the average household income is 15.3% lower than the US average. Geisinger’s mission and vision are to be a model for other developing healthcare systems through continued learning via clinical research [13].

In January 2019, Geisinger implemented an MDLC to facilitate the translation of evidence-based guidelines [2] to the management of high-risk lipid conditions. Patients referred are currently unable to meet cholesterol and triglyceride treatment goals in primary care or cardiology clinics. The Geisinger MDLC is staffed with a cardiologist boarded in lipidology. The genetic counselor and pharmacist both have specialized training in lipid conditions. This clinic meets bi-monthly at one clinic location within the healthcare system. Patients could have traveled from any of Geisinger’s 45 counties within their service area to attend the MDLC.

### Population

Any individual within the Geisinger system diagnosed with or suspected to have a lipid condition can be referred to and seen by the MDLC. A variety of lipid conditions are evaluated and treated in this clinic, including, but not limited to, familial hypercholesterolemia (FH), hypertriglyceridemia, various rare familial dyslipidemias, and other unnamed or undiagnosed dyslipidemias (Table 1) [2].

**Table 1:** Description of lipid conditions and treatment goals

Condition	Description	Treatment goal
Familial hypercholesterolemia (FH)	• Inherited lipid condition	Reduction in LDL-C level
	• Lifelong elevations in LDL cholesterol levels lead to premature ASCVD	
	• Important to test family members	
Hypertriglyceridemia	• Elevated levels of triglycerides	Reduction in triglycerides
	• Cholesterol levels can be normal	
	• At risk for or have had episodes of pancreatitis	
	• Associated with type II diabetes mellitus	
	• May encompass inherited hypertriglyceridemia	
Dyslipidemia	• Elevated levels of triglycerides and/or cholesterol	According to current guidelines, reduction in the lipid that is elevated (cholesterol, triglycerides) [2]
	• Dyslipidemias that are not FH or hypertriglyceridemia	

### Clinical implementation

The purpose of the Geisinger MDLC is to increase uptake of guideline-recommended treatment for all lipid conditions [2]. Prior to implementation of this clinic, individuals with these conditions had to receive specialty multidisciplinary lipid care outside the Geisinger system at locations that required significant travel to urban sites in Pennsylvania for specialized management. Preventive cardiology leadership within the Heart Institute initiated the MDLC and sent out an email to the entire Heart Institute, and Community and Family Medicine providers inviting them to refer patients. The invitation introduced the MDLC, purpose, details on which providers were part of this clinic and how to refer. Table 2 details the MDLC implementation strategy using Proctor’s guidelines for defining and specifying implementation strategies [14] and the template for intervention description and publication checklist.

**Table 2:** Description based on Proctor’s guidelines for specifying implementation strategies: components of the multidisciplinary lipid clinic

Domain	Description
Name it	Creation of new clinical teams (a multidisciplinary lipid clinic)
Define it	A multidisciplinary clinical team that has complementary roles (i.e., diagnosis and treatment) with lipid expertise that is formed to improve patient care
Specify it	
Actors	*Cardiologist*
	*Pharmacist*
	*Genetic counselor*
Actions	*Cardiologist*—evaluates the patient’s symptoms, lifestyle, medications, and past lab results during an initial in-person visit; recommends a treatment plan; orders subsequent testing; requests follow-up visits as needed
	*Pharmacist*—evaluates the patient’s current medications; offers input/suggests changes to medications; performs medication reconciliation; completes medication counseling and education; ensures prior authorizations are submitted
	*Genetic counselor*—evaluates the patient’s past medical and family histories; assesses the patient’s risk; provides pre-test genetic counseling; provides genetic testing result disclosure and post-test genetic counseling; discusses cascade testing of at-risk relatives
Targets of the action	*All clinicians*—have expertise caring for patients with a high-risk lipid condition and knowledge of guideline-recommended treatment for lipid conditions
	*Cardiologist*—diagnosis of lipid conditions, monitors clinical symptoms
	*Pharmacist*—optimizes treatment and follow-up on prior authorizations
	*Genetic counselor*—knowledge of familial cardiovascular conditions, improvement of identification methods for concerning past medical/family history, and reassurance to the patient that the testing results will benefit the patient no matter if the result is positive or negative
Temporality	Patients should be referred as soon as the provider identifies a patient with a high-risk lipid condition who would benefit from the evaluation at the clinic. The initial visit to the clinic should take place as soon as scheduling allows after the patient has been referred. Subsequent visits should be scheduled on an as needed basis.
Dose	*Cardiologist*—once at an hour-long initial visit. Subsequent visits at 6–8 weeks post-initial visit and further if needed. The cardiologist will be available to the patient via phone or through patient portal.
	*Pharmacist*—once at an hour-long initial visit. The pharmacist will be available to the patient via phone or patient portal.
	*Genetic counselor*—once at an hour-long initial visit. The genetic counselor will be available to the patient via phone or patient portal.
Implementation outcomes affected	Uptake of guideline-recommended testing and treatment for high-risk lipid clinic patients; adoption of the clinic among PCPs and other providers; penetration among eligible patients; fidelity to the protocol of the clinic; sustainability of the clinic and its expansion.
Justification	MDLCs improve patient outcomes [3–8]

### Data collection and outcomes measured

This study evaluates the first year of MDLC implementation using the RE-AIM framework. Using a pre/post-study design, clinical outcomes were assessed for all patients 1 year after the implementation of the MDLC. Outcomes were collected from administrative data, and clinical information was collected from the electronic health record (EHR). Two study staff were trained to search the EHR for laboratory measures, medication profiles, and appointment visits and performed chart review after each patient appointment.

*Reach* is measured at the individual level with the numerator defined as the number of patients seen by the MDLC who had both a documented lipid condition on their problem list and had been active patients within the healthcare system (i.e., had a primary care or cardiology visit in 2019). The denominator included those with a problem list diagnosis for a lipid condition. Patients could be referred to the MDLC by any provider using an existing general cardiology outpatient referral for lipid management and were requested to note the MDLC in a comment section. At the time of MDLC implementation, the decision for the system was to use the existing referral rather than to create a new referral specific to MDLC. If the MDLC was not specified in the note, patients could potentially have been seen by any provider with an interest in managing lipid patients.

*Effectiveness* is stratified to create three clinical patient lipid subgroups: FH, hypertriglyceridemia, and dyslipidemia because treatment approaches differ by condition. The final diagnosis was extracted from cardiologist documentation after all relevant information was obtained. The effectiveness measure chosen for this study was the change in lipid levels assessed using a baseline lipid value either from the initial MDLC visit or the most recent lipid panel result prior to the initial MDLC follow-up visit compared with the value from the most recent MDLC visit. Patients without a lipid panel in their health records were documented as having no prior lipid measurements. The most recent lipid panel was recorded as post-MDLC measurement, unless that measurement was the pre-MDLC value. All post-MDLC measurements were at least 1 month after their initial visit date.

*Adoption* has two metrics. Any PCP or cardiologist who saw a patient with a lipid condition documented on their problem list diagnoses in 2019 was included in the analyses. The percent of eligible providers referring to the MDLC was calculated as the number of providers making a referral divided by the total number of eligible providers multiplied by 100. The percent of eligible patients referred per provider—as measured by having a lipid condition in the problem list—was calculated as the number referred over the total number of lipid patients managed by that provider.

*Implementation* is measured at the patient level. The numerator is the number of patients with multiple visits, and the denominator is the number of patients seen in 2019. The percentage of patients who underwent genetic testing and medication use details were reported using descriptive statistics.

*Maintenance*, for this study, is reported as the current and potential for sustaining the MDLC in the future.

### Statistical analysis

Descriptive statistics were used for the demographics of both the study cohort and three subgroups. Continuous variables were analyzed using the Kruskal-Wallis test, and categorical variables were analyzed using a Fisher exact test. We reported the mean ± SD and median (range) of lipid levels for all subgroups and used the Wilcoxon signed-rank test to detect any differences in lipid levels before and after for each subgroup.

## Results

### Reach

Of the 452,748 unique patients who had a visit to the healthcare system in 2019, 32% (143,154/452,748) had a diagnosis of a lipid condition on their problem list. There were 420 referrals received for the MDLC in 2019 out of the 143,154 individuals with a lipid condition. Of those 420 referrals, 3 referenced the “MDLC” specifically and 92 referenced the “lipid clinic.” There were 83 patients scheduled and seen by the MDLC in 2019 (20% of those referred). Of those 83 patients, 1 patient was self-referred, and 4 patients did not have a primary care or cardiology visit in 2019 or lipid condition diagnosis code.

### Effectiveness

Of the 83 patients seen in the MDLC in 2019, 82 were alive at the time of analysis and are presented in the results. Guideline recommendations for management vary based on lipid condition. The MDLC patient population was stratified for analysis based on lipid diagnosis: familial hypercholesterolemia, hypertriglyceridemia, and uncharacterized dyslipidemia. Of those 82 patients, 29% (24/82) had a clinical or genetic diagnosis of (FH), 20% (16/82) had hypertriglyceridemia, and 51% (42/82) had uncharacterized dyslipidemia. Demographics of these populations are described in Table 3.

**Table 3:** Baseline demographics for all patients seen in the MDLC

	All patients, *N* = 82 (ref)	Familial hypercholesterolemia, *N* = 24	Dyslipidemia, *N* = 42	Hypertriglyceridemia, *N* = 16	*P* value
Age in years	56 ± 15	53 ± 16	62 ± 13	45 ± 13	< 0.001
Male	42 (51%)	6 (25%)	25 (60%)	11 (69%)	0.007
BMI	31 ± 8	27 ± 6	32 ± 7	34 ± 10	
Tobacco status					0.436
Never	46 (56%)	16 (67%)	20 (48%)	10 (62%)	
Former	28 (34%)	5 (21%)	18 (43%)	5 (31%)	
Current	8 (10%)	3 (12%)	4 (9%)	1 (7%)	
Problem list diagnosis of CAD	36 (44%)	6 (25%)	26 (62%)	4 (25%)	0.004
Problem list diagnosis of PVD	7 (9%)	2 (8%)	5 (12%)	0	0.502
No reported MIs	67 (82%)	22 (92%)	30 (71%)	15 (94%)	0.243
Number of appointments					0.011
1	41 (50%)	12 (50%)	16 (38%)	13 (81%)	
2	31 (38%)	11 (46%)	19 (45%)	1 (7%)	
3 or more	10 (12%)	1 (4%)	7 (17%)	2 (12%)	

*BMI* body mass index, *CAD* coronary artery disease, *PVD* peripheral vascular disease, *MI* myocardial infarction

#### Familial hypercholesterolemia

Lipid levels were available for comparison before and after MDLC visits in a subset of patients (*n* = 12) and showed a 79-mg/dL reduction in average LDL-C (*P* < 0.001) and reduction in other lipid values (Table 4). Of the 12 patients with pre- and post-values, only 17% (2/12) had an LDL-C less than 100 mg/dL prior to a visit with the MDLC; however, 75% (9/12) met this goal afterwards.

**Table 4:** Lipid levels pre/post-implementation of the MDLC

	Baseline			Post			*P* value
	*N*	Mean ± SD	Median (range)	*N*	Mean ± SD	Median (range)	
Familial hypercholesterolemia (*N* = 12)							
Total cholesterol	12	237 ± 72	247 (133, 361)	12	162 ± 71	150 (86, 346)	< 0.001
HDL-C	12	53 ± 14	53 (25, 79)	12	55 ± 11	57 (33, 73)	0.637
LDL-C	12	163 ± 69	166 (71, 296)	12	87 ± 70	84 (9, 279)	< 0.001
TG	12	118 ± 59	118 (34, 238)	12	96 ± 44	90 (37, 173)	0.077
Non-HDL-C	12	184 ± 69	186 (87, 316)	12	106 ± 73	95 (26, 302)	< 0.001
Dyslipidemia (*N* = 21)							
Total cholesterol	21	213 ± 68	222 (115, 376)	22	168 ± 67	152 (79, 371)	< 0.001
HDL-C	21	44 ± 15	40 (25, 83)	21	46 ± 18	40 (27, 105)	0.223
LDL-C	21	135 ± 67	135 (48, 277)	21	87 ± 56	70 (2, 243)	< 0.001
TG	21	226 ± 182	176 (69, 927)	21	204 ± 199	140 (79, 1002)	0.266
Non-HDL-C	21	169 ± 63	184 (65, 305)	21	121 ± 64	105 (34, 312)	< 0.001
Hypertriglyceridemia (*N* = 4)							
Total cholesterol	4	239 ± 63	264 (146, 284)	4	162 ± 47	148 (121, 230)	NA
HDL-C	4	24 ± 9	27 (11, 31)	4	28 ± 12	32 (11, 37)	NA
LDL-C	4	73 ± 50	84 (4, 121)	3	47 ± 32	65 (10, 65)	NA
TG	4	1123 ± 452	975 (759, 1784)	4	656 ± 613	428 (232, 1536)	NA
Non-HDL-C	4	215 ± 54	236 (135, 254)	4	134 ± 45	112 (110, 202)	NA

*HDL-C* high-density lipoprotein cholesterol, *LDL-C* low-density lipoprotein cholesterol, *TG* triglycerides

#### Hypertriglyceridemia

Lipid levels were available for comparison before and after MDLC visits in a subset of patients (*n* = 4) and showed a 467-mg/dL reduction in average triglycerides (Table 4). Of the 4 patients with pre- and post-values, only a quarter (1/4) had a triglyceride level less than 150 mg/dL prior to a visit with the MDLC; however, 75% (3/4) met this goal afterwards.

#### Uncharacterized dyslipidemia

Lipid levels were available for comparison before and after MDLC visits in a subset of patients (*n* = 21) and showed a 48-mg/dL reduction in average LDL-C (< 0.001) and reduction in other lipid values (Table 4). Of the 21 patients, 38% (8/21) had an LDL-C less than 100 mg/dL prior to a visit with the MDLC; however, 71% (15/21) met this goal afterwards.

### Adoption

Of the 796 active PCP or cardiologists in 2019, 19% (148/796) referred patients to the MDLC. The average percent of eligible patient referrals from active providers was 0.25% (SD 3.75%). There was an average of 35 patient referrals per month (SD 12) to the MDLC. Referrals to MDLC were also received from another 48 providers outside of the targeted cardiologists and PCPs (genetic counselors, pharmacists, critical care providers) or from providers whose patients did not have a diagnosed lipid condition on their problem list.

### Implementation

At the patient level, 50% (41/82) of patients who attended the MDLC had at least one follow-up visit with the MDLC, with 12% (10/82) having two or more follow-up visits in 2019. The 50% (41/82) of patients who only had one visit were more likely to have been seen later in 2019 (October through December). Patient- and provider-level implementation outcomes of genetic testing and prescribing of guideline-recommended treatment are described based on lipid subgroup.

#### Familial hypercholesterolemia

Six of the 24 individuals with FH had prior positive genetic testing for the condition. Those who did not have a prior genetic testing result had testing ordered at the MDLC. Genetic testing ordered through the MDLC (*n* = 18) yielded 5 positive results, 2 variants of unknown significance, and 5 negative results. Six genetic tests are still pending (e.g., order not signed, order expired, active order but sample has not yet been drawn). At the initial MDLC visit, 17 (71%) patients were prescribed at least one medication (e.g., prescribed by some other provider prior to getting to MDLC). Of the 24 patients in the FH subgroup, 1 (4%) had no changes to their medication regimen. This patient did not have any medications pre- or post-MDLC due to pregnancy, which impacts the use of lipid-lowering therapy. The 23 (96%) patients who had a change to their medication regimen fell into one or more of the following categories: 21 patients had an intensification of their medication regimens including starting a medication, increasing the dose of a medication, or addition of a new medication; 1 had a decrease in their dose of a medication; and 10 had other changes including discontinuations or switches within a medication class. Fourteen of the 24 individuals with FH were prescribed medications for which their insurance required a prior authorization. A total of 16 prior authorizations were submitted (3 individuals had prior authorizations submitted for both PCSK9 inhibitors and icosapent ethyl or for two PCSK9 inhibitors). Of the 16 medication prior authorizations submitted by the MDLC, 88% (14/16) were approved (12 for PCSK9 inhibitors and 2 for icosapent ethyl) and 13% (2/16) were denied for PCSK9 inhibitors.

#### Hypertriglyceridemia

None of the 16 individuals attending MDLC diagnosed with hypertriglyceridemia had prior genetic testing. Genetic testing was ordered on 14 of the 16 individuals (88%) and identified 1 positive result for a variant associated with familial lipoprotein lipase deficiency, 2 variants of unknown significance, and 6 negative results; 3 tests are pending, and 2 were not completed. At the initial MDLC visit, 15 (94%) patients were currently prescribed at least one medication to treat their hypertriglyceridemia. Of the 16 patients in the hypertriglyceridemia subgroup, 3 (19%) had no changes to their medication regimen. The 13 (81%) patients who had a change to their medication regimen fell into one or more of the following categories: 10 patients had an intensification of their medication regimens including starting a medication, increasing the dose of a medication, or addition of a new medication; 1 had a decrease in their dose of a medication; and 4 had other changes including discontinuations or switches within a medication class. One person that switched between medication classes was the reconciliation of a drug-drug interaction to prevent harm to the patient. At the time of analysis, 16 (100%) of the 16 patients in the hypertriglyceridemia subgroup after being seen by the MDLC were prescribed a medication for the treatment of hypertriglyceridemia. Three of the 16 individuals with hypertriglyceridemia were prescribed medications by the MDLC for which their insurance required a prior authorization, resulting in a total of 3 prior authorizations submitted. All were approved (2 were for PCSK9 inhibitors and 1 for icosapent ethyl).

#### Uncharacterized dyslipidemia

None of the 42 individuals with dyslipidemia had prior genetic testing for the condition. Genetic testing was ordered by MDLC for 31 (74%) patients and found no positive results, 24 negative results, 6 tests are pending, and 1 was canceled by the patient due to cost. Genetic testing was not ordered for 11 individuals. At the initial MDLC visit, 32 (76%) patients were prescribed at least one medication to treat their dyslipidemia. Of the 42 patients in the dyslipidemia subgroup, 7 (17%) had no changes to their medication regimen. The 35 (81%) patients who had a change to their medication regimen fell into one or more of the following categories: 31 patients had an intensification of their medication regimens including starting a medication, increasing the dose of a medication, or addition of a new medication; 1 had a decrease in their dose of a medication; and 16 had other changes including discontinuations or switches within a medication class. Eighteen of the 42 individuals with dyslipidemia were prescribed medications for which their insurance required prior authorization. A total of 18 prior authorizations were submitted. Of those 18, 16 were approved (for PCSK9 inhibitors), 1 was denied for icosapent ethyl, and 1 had an unknown status for a PCSK9 inhibitor.

### Maintenance

In monthly meetings with the MDLC clinic providers and Heart Institute leadership and administration, we discussed the transition from traditional in-person follow-up visits to telehealth appointments. The rationale for this transition was to improve capacity due to a limited number of available appointments per clinic day and would increase access as individuals would have the opportunity to be seen virtually, either at home or at a clinic site near their home. Additionally, there was a discussion for the need for a telehealth platform, training of schedulers to know where and when to book these appointments, where and how the MDLC providers would join the telehealth visit and obtain access for all providers, and discuss methods for documenting these types of visits. Additionally, a recent pandemic, COVID-19, necessitated the use of telemedicine throughout Geisinger, but this merely accelerated the transition that was already planned for the MDLC.

## Discussion

In our evaluation of the first year after implementing a new care model, we found there are many individuals within our system with lipid conditions, but only a small number have been referred to the MDLC (0.25%), indicating additional implementation strategies may be needed to improve the reach of the MDLC to improve patient care. At present, in the total population of patients with lipid disorders, we do not know how many would be eligible for referral based on medication intolerance or failure to achieve the lipid treatment goal. Only by evaluating this can the true care gap be evaluated and used to develop strategies to expand referrals. We found MDLC improved patient prognosis based on risk stratification, increase in guideline-recommended treatments prescribed, and clinically significant lowering of targeted lipid levels necessary for the prevention of future CVD events. A reduction of 40 mg/dL of LDL-C for individuals with cholesterol conditions is thought to reduce CV events by 20% which is clinically significant and is an accepted intermediate outcome per guidelines [15]. In patients with FH seen through the MDLC, the mean reduction in LDL-C was 79 mg/dL, which is predicted to reduce CV events by 40%. In addition, through MDLC, the number of patients achieving a target of LDL-C below 100 mg/dL increased from 15 to 69%, a more than 4-fold increase.

Based on the referral volume after 1 year of MDLC implementation with referrals from only PCPs and cardiologists, we have shown (1) a need for the MDLC in the Geisinger system, (2) a significant clinical impact on those patients managed by the MDLC, and (3) an enormous care gap with only 0.25% of eligible patients being seen through MDLC reducing the potential impact on CV event prevention.

Barriers to MDLC sustainability included the inability of the MDLC to see all patients that were referred in the first year. Another barrier was not specifying the MDLC in the notes section of the referral by referring providers. Due to these barriers, some patients were not seen by the MDLC but by the designated lipid expert in their corresponding region which led to some frustration by patients and providers who were expecting to be evaluated by a team of lipid experts from multiple disciplines. Facilitators of sustainability included the large cadre of providers who referred patients. The MDLC provided a more refined clinical diagnosis and treatment plan for patients. We believe that this will incentivizes providers to continue to refer severe lipid disorder patients that they are unable to diagnose.

A systematic approach to implementation and continual evaluation of implementation process outcomes and contextual factors is important to implementing these types of programs within healthcare systems. To that end, team members and administrative staff have discussed initiating telehealth appointments as a potential solution for the sustainability of these types of programs in general and the MDLC specifically. By utilizing telehealth, patients would be able to join at their home or drive to a local clinic to connect with the providers at the MDLC located at a central hub. This model could be generalized to other healthcare systems that have large service areas. The COVID-19 pandemic resulted in a system-wide move to telemedicine visits, eliminating many barriers that might have otherwise delayed the implementation.

Other MDLCs have found similar clinical effectiveness outcomes to our clinic [7, 8, 16]. For MDLCs to improve the health of the targeted population, they must first reach the intended individuals. While the existence of an MDLC is helpful, it is not sufficient to ensure utilization of its services. Most of these MDLCs have been implemented within academic medical centers [10] with fewer in healthcare systems, mostly in Veteran’s Affairs [8, 16] or community medical centers [17]. However, it is unclear from these studies the potential impact MDLCs have had on reach within their patient catchment areas due to lack of description or analysis on the process for referrals and number of patients with lipid conditions in these systems. To improve generalizability to other healthcare systems, it is important to understand contextual factors associated with the implementation of a MDLC.

It is widely accepted in implementation science that simply rolling out a new professional guideline or making a new model of care available is insufficient to lead to practice change that will impact patient or population health outcomes [18]. Often, sufficient details are lacking to replicate the implementation strategies utilized for the evidence-based intervention or new care model [14]. Therefore, evaluation of multi-level outcomes related to process and context as well as patient outcomes is necessary when implementing interventions in the real world [19].

Implementation science frameworks have rarely been applied in the field of lipidology or FH [20]. However, their use and benefit have been demonstrated in the implementation of other chronic disease programs such as diabetes control [21]. Using frameworks such as RE-AIM will help lipidologists, and others implementing MDLCs, understand the full impact of their programs and where the barriers and facilitators to access or care exist [22, 23]. In addition, studies like ours will improve the generalizability of these programs to other sites.

This study has a few limitations. Most patients were able to see all providers described within the MDLC; however, there were rare circumstances where one provider might have been unavailable. A specific outpatient referral for the MDLC was not created within our system, though dependent upon the success and volume of this clinic, one can be created in the future. The number of patients seen and followed up in the recommended time frame with the MDLC was limited by clinical location and capacity (one location in the region and twice per month periodicity). However, the Geisinger catchment area spans 45 of Pennsylvania’s 67 counties making it over a 2-h drive for some individuals to reach the current MDLC location. Some individuals seen in the MDLC require multiple visits for complex diagnoses or medication changes, thus limiting the availability of appointment slots for new patients. In addition, we used a pre/post-study design to analyze our data which comes with limitations including the possibility of experiencing a Hawthorne effect. In our analyses, we used a follow-up period defined by time since the clinic was implemented; therefore, patients seen earlier after implementation had longer follow-up intervals than those seen later in the year. Additional analyses conducted farther out from MDLC implementation are needed with larger samples of individuals with a standard follow-up period (e.g., 1-year post-visit). Finally, by limiting to the 1-year period after implementation, some individuals with pending genetic test results in the dyslipidemia category may move into a different category once the test results are received.

## Conclusions

Severe lipid conditions require dedicated care for identification, early intervention, and management. Individuals treated at the Geisinger MDLC show improved clinical outcomes 1 year after MDLC implementation. More attention is needed regarding context, solutions to decrease unseen referrals, appropriate number and duration of MDLC visits, and sustainability. Attention to these things will help determine the impact of MDLC on patient outcomes and improve reach, adoption, sustainability, and replication within other healthcare settings.

## Data Availability

All data generated or analyzed during this study are included in this published article.

## Abbreviations

CVD: Cardiovascular disease
EHR: Electronic health record
FH: Familial hypercholesterolemia
LDL-C: Low-density lipoprotein cholesterol
MDLC: Multidisciplinary lipid clinic
PCP: Primary care provider
RE-AIM: Reach, effectiveness, adoption, implementation, and maintenance
